# The Negative Mental Health Consequences of Social Media Use in South Africa: The Role of Smartphone Addiction

**DOI:** 10.3390/bs16050633

**Published:** 2026-04-23

**Authors:** Tyrone B. Pretorius, Anita Padmanabhanunni

**Affiliations:** 1 Department of Psychology, University of the Western Cape, Bellville 7530, South Africa; 2 Clayton Campus, Monash University, Melbourne 3800, Australia

**Keywords:** social media use integration, smartphone addiction, depression, PTSD, anxiety, mediator

## Abstract

The use of smartphones and social media has become an increasing feature of daily life among university students. Although technology use can offer benefits, growing evidence links heavier engagement to poorer mental health outcomes. This study examined the associations between social media use and indices of psychological distress among South African university students, and it tested whether smartphone addiction represents a pathway linking social media use to distress. Participants (*n* = 491) were students who completed the Social Media Use Integration Scale, the Smartphone Application-Based Addiction Scale, the Center for Epidemiological Studies Depression Scale-10, the Posttraumatic Stress Disorder Checklist for DSM-5, the Beck Hopelessness Scale-9, and the trait scale of the State-Trait Anxiety Inventory-5. Mediation analyses with the PROCESS macro was conducted to examine smartphone addiction as a possible pathway between social media use and indices of psychological distress. The results of the mediation analysis indicated that social media use and smartphone addiction had significant positive direct effects on depression, PTSD, and anxiety, but not on hopelessness. In addition, social media use had significant indirect effects via smartphone addiction on depression, PTSD, and anxiety, pointing to the partial mediating role of smartphone addiction. The results highlight the importance of incorporating targeted support within student mental health services. Interventions aimed at reducing distress may benefit from targeting problematic smartphone engagement alongside broader efforts to promote healthier social media practices.

## 1. Introduction

Among university students the use of smartphones and social media have become an increasing feature of daily life. Smartphones provide near-continuous access to social networking platforms, messaging applications, and online communities, enabling students to remain connected to peers, academic networks, and news in real time. Several studies suggest that smartphones are predominantly used for social media activities and, for many students, social media use is integrated into everyday routines and shapes how relationships are initiated, maintained, and evaluated (Salari et al., 2025). Social media, in this context, refers to online platforms or applications (e.g., Facebook, Instagram, and TikTok) that allow individuals to create, share and interact with content, thereby fostering social connections and a sense of community (Kaplan & Haenlein, 2010).

Research on social media use and psychological wellbeing have produced mixed results. Some studies have reported that social media use can strengthen existing relationships, help maintain contact across distance, facilitate peer support, and create opportunities for belonging among individuals who may feel isolated in offline settings (Bekalu et al., 2019; Helliwell et al., 2026; Taylor et al., 2022). Prior evidence from studies on university student populations have also suggested that social media use may be associated with better outcomes for students with lower self-esteem and lower life satisfaction (Ellison et al., 2007; Steinfield et al., 2008). In their systematic review, Seabrook et al. (2016) concluded that social media use was associated with lower levels of loneliness and higher self-esteem and life satisfaction. However, some scholars have cautioned that this evidence should be interpreted carefully, given that many of these studies rely on cross-sectional designs, self-report measures, and effects that are relatively small in magnitude (O’Day & Heimberg, 2021).

Furthermore, positive outcomes have been linked to the motivations underlying its use and the manner in which social media is used, especially whether use is active or passive (Godard & Holtzman, 2024). Active social media use, which includes direct communication and reciprocal interaction, tends to be associated with greater social connectedness and perceived support (Frison & Eggermont, 2016). In contrast, passive use, characterized by browsing or monitoring others without meaningful interaction, has more often been associated with negative social comparison and poorer well-being (Valkenburg et al., 2022). This suggests that the psychological effects of social media may depend less on use itself than on the quality and form of engagement.

Although social media may promote connection and belonging, engagement with these platforms is typically embedded within broader patterns of smartphone use. Consequently, the possible benefits of social media must be considered alongside growing evidence that heavier or more immersive smartphone engagement is associated with poorer health-related outcomes (Alonzo et al., 2021; Galanis et al., 2025; Y. Lee et al., 2022; Lekgothoane & Kaminer, 2024). At the maladaptive end of this continuum, problematic smartphone use, or smartphone addiction refers to a dysfunctional pattern of smartphone engagement characterized by compulsive checking, difficulty controlling usage, and use in inappropriate or potentially dangerous situations (e.g., while driving) (Csibi et al., 2018; Montag et al., 2021). This pattern is associated with adverse consequences for psychological and social well-being, including impaired academic and occupational functioning (Busch & McCarthy, 2021; Pivetta et al., 2019).

Heavy engagement with social media and social networking platforms may represent a key pathway through which problematic smartphone use develops (Koç & Turan, 2021; Marino et al., 2021). However, smartphone addiction is a broader construct that captures dysfunctional attachment to the device itself and the compulsive use of multiple smartphone-enabled functions (e.g., messaging, browsing, gaming, streaming, etc.), rather than social media use alone (Rozgonjuk et al., 2022).

Among adolescents and young adults, smartphone addiction has consistently been linked to higher levels of internalizing mental health conditions (e.g., anxiety, depression, and hopelessness), and more frequent sleep difficulties (Ratan et al., 2021; Stanković et al., 2021). One proposed explanation is that excessive smartphone engagement can displace face-to-face interactions and hinder the development and maintenance of supportive offline relationships (Verduyn et al., 2021). Studies provide some support for this argument at the within-person level, indicating that periods of greater use may coincide with lower levels of direct interaction, even if this pattern is less evident between individuals (Kross et al., 2013; Verduyn et al., 2021). However, the literature also points to the paradoxical nature of social connection online in that digital platforms may enhance connection and belonging in some contexts while contributing to isolation and reduced well-being in others (Lopes et al., 2022; Verduyn et al., 2021). Furthermore, cyberbullying and negative responses to social media posts have been identified as contributing factors to psychological distress (H. Y. Lee et al., 2020).

Scholars have highlighted the role of online self-presentation processes in the association between problematic smartphone use and psychological distress (Skogen et al., 2021; Stebleton et al., 2022). Online self-presentation refers to the manner in which individuals control and manage how they are potentially perceived by others (Wilson et al., 2024). Since smartphones provide continual access to social media platforms and immediate feedback about one’s visibility and social standing, excessive use may intensify concerns about how one is perceived by others online (Wong & Hamza, 2025). This can include curating self-related content, closely monitoring reactions from others, and becoming increasingly sensitive to social evaluation. These dynamics may, in turn, promote greater self-scrutiny and social comparison, particularly when individuals evaluate themselves against idealized portrayals of others (Skogen et al., 2021). Such processes have been linked to greater negative affect, lower self-esteem, and elevated psychological distress (Hawes et al., 2020; Skogen et al., 2021; X. Wang et al., 2025).

Although depression and anxiety are the most frequently measured outcomes in relation to smartphone addiction, research has also suggested an association with post-traumatic stress disorder (PTSD) (Contractor et al., 2017; Milam et al., 2026; Padmanabhanunni & Pretorius, 2025). In this regard, smartphone addiction may function as an avoidant coping strategy to dampen distressing emotions or distract from intrusive memories (Contractor et al., 2019). While such use may provide short-term relief, it can inadvertently maintain or exacerbate PTSD symptoms by reinforcing avoidance and disrupting sleep. Moreover, persistent connectivity can increase exposure to distressing or trauma-related content, further amplifying stress responses in vulnerable individuals (Gong & Li, 2025; Milam et al., 2026).

The I-PACE (Interaction of Person–Affect–Cognition–Execution) model, originally developed in the addictions literature and later adapted to explain problematic internet and technology use, has been widely used to understand the development and maintenance of smartphone addiction (Ge et al., 2023; Mehmood et al., 2021). The model proposes that problematic technology use arises through interactions between predisposing person factors, affective responses, cognitive processes, and executive functioning (Brand et al., 2016). In the context of smartphone addiction, person-level vulnerabilities may include factors such as prior trauma exposure or psychological distress; affective processes may include stress reactivity or negative mood states; cognitive processes may involve expectancies that smartphone use will provide relief, distraction, or social reward; and executive functioning refers to the ability to regulate urges and disengage from rewarding digital cues (C.-W. Chang et al., 2023; Sánchez-Fernández & Borda-Mas, 2023). From this perspective, smartphone addiction can be understood as the outcome of repeated technology use that becomes increasingly reinforcing for vulnerable individuals, particularly when emotion regulation and inhibitory control are compromised (Mehmood et al., 2021).

In sum the literature suggests that the relationship between smartphone use and psychological distress is complex and conditional rather than uniform (Taylor et al., 2022). General smartphone use may serve adaptive interpersonal functions, especially when it supports communication, belonging, and access to support (Bekalu et al., 2019; Godard & Holtzman, 2024). However, more immersive, compulsive, or emotionally dependent forms of engagement appear more likely to be associated with poorer mental health outcomes (Lopes et al., 2022). These issues are especially salient in university student populations, where developmental transitions, academic pressures, and vulnerability to mental health difficulties may heighten both reliance on digital connection and susceptibility to its harms (Salari et al., 2025).

This study focused on South African university students, a group that has been consistently identified as being at an elevated risk for adverse mental health outcomes (Bantjes et al., 2022; Coetzee et al., 2024; Padmanabhanunni & Wiid, 2022). South African research on social media use, smartphone addiction, and related mental health outcomes remains limited, and the available evidence points to both beneficial and adverse associations. Some studies have captured the potential benefits of social media use among adolescents (Shava & Chinyamurindi, 2018) and university students (Lekgothoane & Kaminer, 2024), including information-sharing and socialization practices. Concerns about problematic patterns of use are, however, also emerging. For example, in a study of university students, 38% of the participants showed signs of problematic social media use, alongside similarly high levels (55%) of problematic smartphone use (Mostert, 2025). In line with this, among adults, other work has reported associations between excessive social media use, sleep difficulties, and depression (De Doncker & McLean, 2022). Extending beyond South Africa, evidence from sub-Saharan African samples has also indicated a relationship between problematic smartphone use and depression (Nkwo et al., 2025).

The current study aimed to extend the literature on social media integration, smartphone addiction, and psychological distress among South African university students by examining the associations between social media integration and symptoms of anxiety, depression, hopelessness, and PTSD. The study also examined whether smartphone addiction functioned as an indirect pathway through which social media integration was associated with these indicators of psychological distress.

Psychological distress is a broad construct that refers to emotional suffering and impaired psychological functioning, and commonly includes experiences such as sadness, anxiety, tension, and difficulty coping with stress (Drapeau et al., 2012; Ridner, 2004). In the present study, it was operationalized using depression, anxiety, PTSD symptoms, and hopelessness as these represent distinct but related forms of internalizing distress.

Depression is characterized by low mood, loss of interest, and negative self-evaluation; anxiety by excessive worry, fear, and physiological arousal; and PTSD by trauma-related symptoms such as intrusive memories, avoidance of reminders of the trauma, hyperarousal, alterations in cognition and emotional dysregulation (American Psychiatric Association, 2022). Hopelessness refers to a cognitive state characterized by negative expectations about the future, a sense of helplessness, and the belief that one is unable to bring about positive change or prevent adverse outcomes (Beck et al., 1974). Although conceptually distinct, these conditions are commonly grouped under the broader umbrella of psychological distress because each reflects significant emotional strain and disruptions in well-being and functioning (Drapeau et al., 2012). In the current study, it was hypothesized that:

**H1.**  *Higher levels of social media integration would be positively associated with higher levels of smartphone addiction.*

**H2.**  *Higher levels of social media integration would be positively associated with higher levels of anxiety, depression, hopelessness, and PTSD symptoms.*

**H3.**  *Higher levels of smartphone addiction would be positively associated with higher levels of anxiety, depression, hopelessness, and PTSD symptoms.*

**H4.**  *Smartphone addiction would partially mediate the association between social media integration and the indicators of psychological distress, such that higher social media integration would be associated with greater smartphone addiction, which in turn would be associated with higher levels of anxiety, depression, hopelessness, and PTSD symptoms.*

## 2. Materials and Methods

### 2.1. Participants and Procedure

Participants (*n* = 491) were students at a university in the Western Cape province of South Africa. Google Forms were used to create an electronic questionnaire consisting of the instruments described in the Measures section. The electronic link and an invitation to participate in the study were distributed to 4000 registered students by the office of the Registrar of the University.

A sensitivity analysis, conducted with the package “pwr2ppl” (Aberson, 2019) in R software version 4.3.1 (R Development Core Team, 2020) assuming medium effect sizes, alpha = 0.05, and one mediator, found that a sample of 155 participants provides 80% power, while power achieved with the current sample of 491 was 100%.

Most of the sample were women (64.8%) and registered for undergraduate studies (92.5%). While participants came from all provinces in South Africa, slightly more than half (60.3%) were drawn from the Western Cape (32.8%) and the Eastern Cape (27.5%) provinces. The mean age of the sample was 21.22 years (*SD* = 3.52).

### 2.2. Measures

In addition to a brief demographic questionnaire, the participants completed the Social Media Use Integration Scale (SMUIS: Jenkins-Guarnieri et al., 2013), the Smartphone Application-Based Addiction Scale (SABAS: Csibi et al., 2018), the Center for Epidemiological Studies Depression Scale-10 (CESD-10: Andresen et al., 1994), the Posttraumatic Stress Disorder Checklist for DSM-5 (PCL-5: Blevins et al., 2015), the Beck Hopelessness Scale-9 (BHS-9: Balsamo et al., 2020), and the trait scale of the State-Trait Anxiety Inventory-5 (STAI-T5: Zsido et al., 2020).

The SMUIS is a 10-item measure assessing the extent to which social media is integrated into a person’s daily life, as well as the degree of emotional connection and investment in social media. The SMUIS has a two factor structure comprising Social Integration and Emotional Connection, and Integration into Social Routines (ISR) (Jenkins-Guarnieri et al., 2013). Only the total scale was used in the current study due to the low reliability of ISR scores (0.67). An example of an item of the SMUIS is “I get upset when I can’t log on to social media (e.g., Instagram, Facebook, TikTok, etc.).” Responses are made on a 6-point scale ranging from 1 (strongly disagree) to 6 (strongly agree), and item scores were summed to yield an aggregate total score, with higher scores indicating greater social media integration and emotional connection.

Although the SMUIS is often administered with reference to a specific social networking site (e.g., Instagram), the broader term “social media” was used in the present study to reflect the focus on participants’ overall social media integration and emotional connection across platforms, rather than use of any one specific site. The authors of the SMUIS reported an estimate of internal consistency for SMUIS scores of 0.91 in a sample of emerging adults and correlations between SMUIS scores and other measures of social media use provided evidence of convergent validity (Jenkins-Guarnieri et al., 2013). In South Africa, Maree (2017) examined the psychometric properties of the SMUIS in relation to Facebook and LinkedIn use and reported estimates of internal consistency of 0.89 and 0.79 for Facebook and LinkedIn use, respectively. The internal consistency of the total SMUIS score in the present sample was acceptable (α = 0.87).

The SABAS is a measure of smartphone addiction and consists of six items that are scored on a 6-point scale ranging from 1 (strongly disagree) to 6 (strongly agree). It is generally treated as a unidimensional measure of smartphone addiction (Csibi et al., 2018). An example of an item from the SABAS is “preoccupying myself with my smartphone is a way of changing my mood (I get a buzz, or I can escape or get away, if I need to).” Scores are summed to produce a total score, with higher scores indicating greater smartphone addiction. The authors of the English version of the SABAS reported an estimate of internal consistency of 0.81 for SABAS scores and also provided evidence for congruent validity (Csibi et al., 2018). We could not find any study that validated the use of the SABAS in South African samples. The internal consistency of the SABAS in the present sample was acceptable (α = 0.81).

The CESD-10 is a 10-item short form version of the original 20-item CES-D (Radloff, 1977) and is used to screen for symptoms of depression. The scale is typically treated as a unidimensional measure of depression severity (Andresen et al., 1994). The CESD-10 is scored on a four-point scale that ranges from 0 (rarely or none of the time) to 3 (most or all of the time). Item responses are summed to obtain a total score, with higher scores reflecting higher levels of depressive symptoms. An example of an item of the CESD-10 is “I had trouble keeping my mind on what I was doing”. Andresen et al. (1994) who shortened the CES-D reported test–retest correlations of 0.71 for the CESD-10 and a positive correlation with mental health status and a negative correlation with positive effect provided evidence for validity. In South Africa, Padmanabhanunni and Pretorius (2024b) validated the CESD-10 and reported satisfactory estimates of internal consistency (α = 0.84, ω = 0.85). In the present sample, the CESD-10 showed acceptable internal consistency (α = 0.76).

The PCL-5 is a 20-item measure that assesses the 20 DSM-5 symptoms of PTSD. While typically conceptualized as measuring the four symptom clusters of PTSD, a South African study found support for a unidimensional factor structure (Padmanabhanunni & Pretorius, 2024b). The 20 items are responded to on a 5-point scale that ranges from 0 (not at all) to 4 (extremely) and high scores reflect high levels of PTSD. Scores are summed to yield an aggregate PTSD symptom severity score, with higher scores indicating greater PTSD symptom severity. An example of an item of the PCL-5 is “how much were you bothered by repeated, disturbing dreams of the stressful experience?”. The authors of the PCL-5 reported strong estimates of internal consistency of 0.94 and 0.95 in two separate samples and provided evidence for convergent and discriminant validity (Blevins et al., 2015). In South Africa, Padmanabhanunni and Pretorius (2024b), reported similar satisfactory estimates of internal consistency (α and ω = 0.94). The internal consistency of the PCL-5 total score in the present sample was excellent (α = 0.94).

The BHS-9 is a 9-item short form version of the original 20-item BHS (Beck et al., 1974) that was designed to assess feelings of hopelessness and pessimism. Prior work has supported the use of the BHS-9 as an essentially unidimensional measure (Padmanabhanunni & Pretorius, 2024a). Items are scored dichotomously (true/false), and responses are summed to produce a total hopelessness score, with higher scores indicating greater hopelessness. An example of an item of the BHS-9 is “There’s no use in really trying to get anything I want because I probably won’t get it”. Balsamo et al. (2020), who shortened the BHS, reported satisfactory estimates of internal consistency for BHS scores (alpha and Mokken scale reliability = 0.86). In South Africa a study with a non-clinical sample found that the BHS-9 was essentially unidimensional and reported sound estimates of internal consistency for BHS-9 scores with α and ω indices of 0.83 and a Mokken scale reliability of 0.85 (Pretorius & Padmanabhanunni, 2024). The internal consistency of the BHS-9 in the present sample was acceptable (α = 0.78).

The STAI-T5 is a 5-item shortened version of the original 20-item STAI-T (Spielberger, 1985) that was designed to measure chronic anxiety and is unidimensional in nature (Pretorius & Padmanabhanunni, 2023). Participants respond to the five items using a 4-point scale that ranges from 1 (not at all) to 4 (very much so). Item scores are summed to yield a total score, with higher scores indicating greater anxiety. Zsido et al. (2020), who shortened the STAI, reported an alpha coefficient of 0.86 for the STAI-T5 scores and provided evidence of external validity. In South Africa, Pretorius and Padmanabhanunni (2023) reported satisfactory reliability coefficients (α and ω = 0.88) and provided evidence of convergent validity. In the present sample, the STAI-T5 showed good internal consistency (α = 0.89).

### 2.3. Ethics

Ethical approval to undertake the study was granted by the Biomedical Science Research Ethics Committee of the University of the Western Cape (Ethics reference: BM23/10/8, December 2023) and the study was conducted according to the guidelines of the Declaration of Helsinki. Participants provided informed consent on the landing page of the electronic link and participation was entirely voluntary. No incentives were offered for participation.

### 2.4. Data Analysis

All items in the electronic questionnaire were flagged as mandatory and participants could not proceed to a subsequent page unless all items were responded to on the current page; thus, there were no missing data. IBM SPSS for Windows version 31 (IBM Corp., Armonk, NY, USA) was used to obtain descriptive statistics (means and standard deviations), distribution indices (skewness and kurtosis), intercorrelations between study variables (Pearson’s *r*), and estimates of internal consistency (Cronbach’s alpha). SPSS by default provides excess kurtosis (kurtosis − 3) and variables are considered to be approximately normally distributed if skewness and excess kurtosis ranged between −2 and +2 (Hair et al., 2021). For research purposes (as opposed to diagnostic use) measurement scores are considered to be reliable if α ≥ 0.70 (DeVon et al., 2007). Correlation coefficients were also evaluated in terms of effect size where *r* < 0.1 = negligible, r between 0.1 and 0.3 = small effect, 0.3 to 0.5 = medium effect, and >0.5 = large effect (Cohen, 2013).

The PROCESS macro in SPSS (model 4) was used to examine the direct and indirect effects (via smartphone addiction) of social media use on indices of psychological distress. In the PROCESS analysis, social media use was the independent variable, smartphone addiction the mediator, and depression, PTSD, hopelessness, and anxiety in turn the dependent variables. In the PROCESS analysis the significance of the indirect effect (mediating effect) was evaluated using 95% bootstrapped confidence intervals. Since PROCESS is a regression-based approach to mediation analysis, we examined the assumption of linearity through visual inspection of scatterplots of the relationships between social media use and the indices of psychological distress. Multicollinearity was examined using the variance inflation factor (VIF) and a VIF > 5 is considered reflective of multicollinearity and indicates that the independent variables are to highly correlated with each other.

## 3. Results

Table 1 reports the intercorrelations between study variables, descriptive statistics (means and standard deviations), distribution indices (skewness and kurtosis) and estimates of internal consistency.

The skewness (−0.18 to 0.83) and kurtosis (−0.99 to 0.28) values in Table 1, were within the acceptable range of −2 to +2, reflecting that the data are approximately normally distributed. The estimates of internal consistency in Table 1 range between 0.76 and 0.94, indicating satisfactory reliability of scores for all scales.

Table 1 further shows that social media use was significantly positively associated with smartphone addiction (*r* = 0.43, *p* < 0.001, medium effect size), depression (*r* = 0.18, *p* < 0.001, small effect size), PTSD (*r* = 0.35, *p* < 0.001, medium effect size), and anxiety (*r* = 0.35, *p* < 0.001, medium effect size), but not associated with hopelessness. These obtained correlations would indicate that high levels of social media use are associated with high levels of smartphone addiction and psychological distress.

Similarly, smartphone addiction was significantly positively associated with depression (*r* = 0.17, *p* < 0.001, small effect size), PTSD (*r* = 0.40, *p* < 0.001, medium effect size), and anxiety (*r* = 0.37, *p* < 0.001, medium effect size), but not with hopelessness. Therefore, high levels of smartphone addiction are associated with high levels of psychological distress.

All of the assumptions of the regression-based mediation analysis were satisfied: scatterplots indicated linear relationships between social media use and the indices of psychological distress, and VIF values were all <5 and ranged between 1.23 and 2.25. Table 2 indicates the direct effects of social media use and smartphone addiction, and the indirect effects of social media use on the indices of psychological distress.

Table 2 indicates that social media use had both significant direct effects (depression: β = 0.13, *p* = 0.01; PTSD: β = 0.13, *p* < 0.001; anxiety: β = 0.13, *p* < 0.001) and indirect effects via smartphone addiction on depression (β = 0.05, 95% CI [0.00, 0.06]), PTSD (β = 0.13, 95% CI [0.15, 0.32]), and anxiety (β = 0.12, 95% CI [0.03, 0.07). In each case, the inclusion of smartphone addiction reduced, but did not eliminate, the direct association between social media use and psychological distress. Specifically, the direct effect decreased from 0.18 to 0.13 for depression, from 0.35 to 0.22 for PTSD, and from 0.35 to 0.23 for anxiety. This pattern indicates positive partial mediation, meaning that higher levels of social media use were associated with greater smartphone addiction, which in turn was associated with higher levels of depression, PTSD, and anxiety. At the same time, social media use remained directly associated with these outcomes, suggesting that smartphone addiction explains part, but not all, of the relationship between social media use and psychological distress. Table 2 further demonstrates that smartphone addiction had significant direct effects on depression (β = 0.12, *p* = 0.01), PTSD (β = 0.31, *p* < 0.001), and anxiety (β = 0.26, *p* < 0.001), independent of social media use.

**Figure 1:**
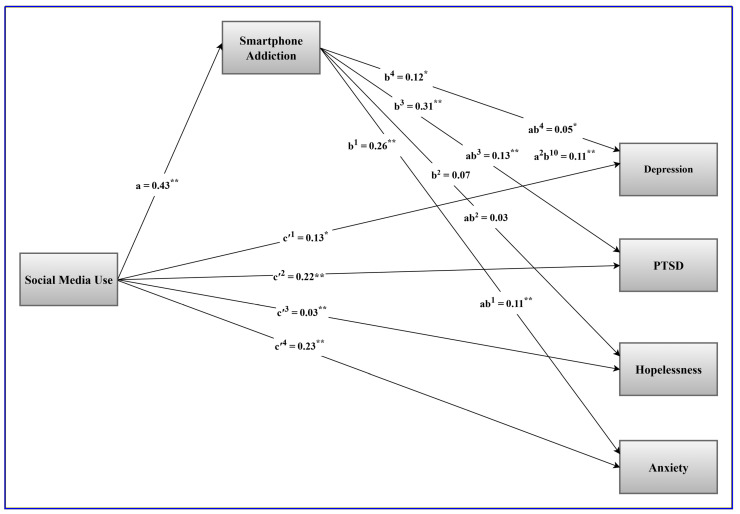
Visual presentation of the mediation model showing the indirect effects of social media use on indices of psychological distress via smartphone addiction. *Note.* c′^1^ to c′^4^ = the direct effects of social media use on the indices of psychological distress, a = the association between social media use and smartphone addiction, b^1^ to b^4^ = the associations between smartphone addiction and indices of psychological distress, ab^1^ to ab^4^ = the indirect effects of social media use on indices of psychological distress via smartphone addiction. * *p* < 0.05, ** *p* < 0.001.

The mediation model is visually presented in Figure 1.

In summary: smartphone addiction partially acted as the pathway through which social media integration impacted indices of psychological distress. In addition, social media integration also had direct effects on the indices of psychological distress. Apart from acting as a pathway, smartphone addiction had direct effects on indices of psychological distress. Social media use and smartphone addiction had no direct or indirect effects on hopelessness.

## 4. Discussion

The present study examined the associations between social media integration and indices of psychological distress, and specifically the role of smartphone addiction in this relationship. There were several significant findings. First, high levels of social media integration were significantly associated with smartphone addiction, anxiety, depression and PTSD but not with hopelessness. This finding is consistent with prior research indicating that heavy engagement with social media may be linked to problematic smartphone use (Koç & Turan, 2021). The association between excessive social media use and anxiety and depression, respectively has been ascribed to upward social comparison (Yang et al., 2023). Social media platforms frequently expose users to highly curated depictions of peers’ achievements, lifestyles, and appearances. When individuals evaluate themselves against these selectively positive portrayals, they may be more likely to appraise themselves as falling short. Over time, repeated upward comparisons can contribute to negative self-evaluation and heightened worry about social standing, which can contribute to mood disturbances (Yang et al., 2023). These effects may be amplified in university contexts where identity formation, belonging, and performance concerns are particularly salient for students (Bozkurt & Tu, 2016; Stebleton et al., 2022). Furthermore, systematic review evidence indicates that the relationship between problematic social media use and depression or anxiety is often bidirectional, with digital engagement potentially contributing to distress while pre-existing distress may also increase reliance on social media and smartphones (Lopes et al., 2022). Recent work has also highlighted the substantial heterogeneity in social media effects, arguing that these effects differ across users,, cultures and contexts (Zhou et al., 2021). Accordingly, the present findings should be interpreted as reflecting one pattern of association within this sample rather than a universal pathway.

In relation to PTSD, one plausible explanation is that social media platforms can increase the likelihood of encountering trauma-related material. This exposure may trigger trauma memories and intensify PTSD symptoms (e.g., intrusive re-experiencing). It is also plausible that excessive social media use may function as a form of avoidant coping, providing short-term distraction from distressing memories and emotions and this may account for the association with PTSD (Milam et al., 2026). The absence of an association with hopelessness is noteworthy as it contrasts with prior research in this area (Li et al., 2024; Talan et al., 2023). Hopelessness reflects a set of negative expectations about the future that may be less directly shaped by day-to-day patterns of social media engagement compared to mood-related and PTSD symptoms. This may account for the current finding.

Second, smartphone addiction was significantly positively associated with depression, PTSD, and anxiety, but not with hopelessness. This pattern is partly consistent with the broader literature, which has more reliably linked problematic smartphone use to proximal forms of psychological distress such as depressive symptoms, anxiety, sleep problems, and arousal-related difficulties (Carter et al., 2024; Milam et al., 2026). By contrast, hopelessness may operate somewhat differently. As conceptualized in the cognitive literature, hopelessness reflects relatively stable negative expectations about the future and perceptions of limited control over desired outcomes, rather than only immediate affective distress (Beck et al., 1974). In the digital-use literature, hopelessness has been associated with mobile phone addiction in some adolescent samples, particularly in more vulnerable groups and in conjunction with maladaptive emotion-focused coping (Li et al., 2024). Among university students, hopelessness has also been associated with cyberloafing, that is, the use of an organization’s internet resources for non-work-related activities (Talan et al., 2023). However, the same study found that smartphone addiction was not directly related to higher hopelessness or lower life satisfaction. In light of this mixed evidence, the null finding in the present study should be interpreted cautiously.

The absence of an association with hopelessness may reflect the phenomenology of the construct. Hopelessness is relatively trait-like and longitudinally stable, and is defined by future-oriented cognitive appraisals (i.e., beliefs that one lacks control over outcomes and expectations of a negative future) (Beck et al., 1974). In contrast, smartphone addiction is more closely linked to proximal, state-like disruptions in daily functioning (e.g., sleep disruption, physiological hyperarousal, etc.). These processes are more directly aligned with fluctuations in mood and arousal symptoms (e.g., anxiety, depressive symptoms, and PTSD-related hyperarousal) than with broader, generalized beliefs. Accordingly, smartphone addiction may be more consistently related to immediate internalizing symptoms, such as anxiety and depressive affect, than to hopelessness specifically. Certain studies have also noted a bidirectional relationship between smartphone addiction and adverse mental health outcomes

Third, smartphone addiction partially mediated the association between social media integration and indices of psychological distress. This result is conceptually consistent with the notion that repeated social media engagement may strengthen habitual checking through intermittent reinforcement (e.g., notifications and social feedback) (J. Wang & Wang, 2025). Over time, such reinforcement shifts smartphone use from intentional engagement to more automatic, cue-driven behavior, where notifications or stress trigger use with little deliberation. Heavy reliance on the smartphone for short-term relief (e.g., distraction from distress) may displace more adaptive coping and create a cycle in which problematic use amplifies psychological distress (K. Chang et al., 2022; Koç & Turan, 2021).

The findings have both theoretical and practical implications. Theoretically, the results lend support to I-PACE-informed models by demonstrating that smartphone addiction functions as a meaningful pathway through which integrated social media use relates to psychological distress. The inclusion of PTSD symptoms extends this literature beyond the typical focus on depression and anxiety, underscoring that problematic digital engagement may also be relevant to trauma-related symptomatology in student populations where trauma exposure is a salient contextual factor.

The current study is conceptually aligned with the I-PACE model in that social media integration reflects a form of rewarding and emotionally salient digital engagement, whereas smartphone addiction reflects the maladaptive technology-use outcome (Ge et al., 2023; Mehmood et al., 2021). In the present model, the pathway from social media integration to smartphone addiction maps onto the I-PACE proposition that repeated engagement with rewarding digital activities may, under certain affective and cognitive conditions, develop into problematic use. The subsequent paths from smartphone addiction to anxiety, depression, and PTSD symptoms are consistent with the idea that problematic technology use is associated with impaired emotional functioning and psychological distress (Mehmood et al., 2021).

Practically, these findings indicate that interventions aiming to mitigate the mental health burden associated with social media use among university students may benefit from targeting problematic smartphone use patterns rather than focusing solely on reducing time spent on social media. Psychoeducation on the negative effects of excessive smartphone use and brief, skills-based interventions that strengthen self-regulation and reduce cue-driven checking may be beneficial in student populations.

Several limitations should be considered when interpreting the findings. First, the study used a cross-sectional design, which precludes causal inference regarding the directionality of the associations observed. Although the model was specified such that smartphone addiction partially mediated the association between social media integration and psychological distress, it is equally plausible that distress contributes to greater reliance on social media and smartphones for coping or avoidance, or that bidirectional influences operate over time. Longitudinal and experimental designs are therefore needed to clarify temporal ordering and reciprocal pathways. Second, all constructs were assessed using self-report measures, which introduces the possibility of self-selection, shared method variance, recall bias, and social desirability effects. Future studies would benefit from including objective indicators, such as digital trace data or screen-time measures, alongside self-report instruments. Third, the sample comprised mostly female university students and was drawn from a single geographic area, which may limit the generalizability of the findings to more diverse socioeconomic and cultural contexts. The findings should therefore be interpreted with caution outside this context. Fourth, the study focused on self-reported indicators of psychological distress rather than clinical diagnoses, and the observed associations should not be interpreted as evidence of psychiatric disorder. Fifth, although PROCESS analysis was useful for examining indirect associations, it is possible that other unmeasured factors, such as personality traits, pre-existing mental health difficulties, trauma exposure, coping style, or social support, may also have influenced the observed relationships. Fifth, the measure of social media engagement captured not only use, but also the extent to which social media was integrated into daily routines and emotionally salient to participants. This has implications for interpretation, as the associations observed may be driven less by use itself than by the degree to which social media is embedded in participants’ routines and social lives. Finally, the mediation analysis was conducted using observed total scores. As a result, measurement error was not modeled directly, and the estimated paths may therefore be attenuated or less precise than those obtained from latent variable models. The use of total scores may also obscure potentially different effects across subdimensions of the constructs. Future studies should consider latent variable modeling to better account for measurement error and the multidimensional nature of the measures.

## 5. Conclusions

This study examined the associations between social media use integration, smartphone addiction, and psychological distress among South African university students. The findings extend the literature by demonstrating the relevance of smartphone addiction as a possible pathway linking social media use to psychological distress. From an applied perspective, the results indicate that prevention and support efforts may benefit from focusing on problematic smartphone use processes rather than emphasizing the frequency of social media use alone.

## Figures and Tables

**Table 1:** Intercorrelations, descriptive statistics, distribution indices and estimates of internal consistency.

Variables	1	2	3	4	5	6
1. Social media use	—					
2. Smartphone addiction	0.43 **	—				
3. Depression	0.18 **	0.17 **	—			
4. PTSD	0.35 **	0.40 **	0.47 **	—		
5. Hopelessness	0.06	0.08	0.43 **	0.21 **	—	
6. Anxiety	0.35 **	0.37 **	0.47 **	0.73 **	0.20 **	—
$\bar{X}$	32.88	21.92	14.05	32.57	2.60	11.91
*SD*	10.69	6.64	5.80	18.62	2.39	4.34
Skewness	0.40	−0.18	0.20	0.12	0.83	0.06
Kurtosis	−0.51	−0.57	−0.50	−0.61	−0.28	−0.99
α	0.87	0.81	0.76	0.94	0.78	0.89

** *p* < 0.001.

**Table 2:** The direct and indirect effects resulting from the mediation analysis.

Type of Effect	Effect	B	SE	95% CI	β	*p*
Direct effects	Social media use → Depression	0.07	0.03	[0.02, 0.12}	0.13	0.01
	Social media use → PTSD	0.38	0.08	[0.22, 0.52]	0.22	<0.001
	Social media use → Hopelessness	0.01	0.01	[−0.02,0.03]	0.03	0.51
	Social media use → Anxiety	0.10	0.02	[0.06, 0.13]	0.23	<0.001
	Smartphone addiction → Depression	0.10	0.03	[0.02, 0.19]	0.12	0.01
	Smartphone addiction → PTSD	0.86	0.13	[0.61, 1.11]	0.31	<0.001
	Smartphone addiction → Hopelessness	0.02	0.02	[−0.01, 0.06]	0.07	0.18
	Smartphone addiction → Anxiety	0.17	0.03	[0.11, 0.23]	0.26	<0.001
Indirect effects	Social media use → Smartphone addiction → Depression	0.03	0.01	[0.00, 0.06]	0.05	— ^a^
	Social media use → Smartphone addiction → PTSD	0.23	0.04	[0.15, 0.32]	0.13	—
	Social media use → Smartphone addiction → Hopelessness	0.01	0.01	[−0.00, 0.02]	0.03	—
	Social media use → Smartphone addiction → Anxiety	0.05	0.01	[0.03, 0.07]	0.11	—

^a^ Significance of indirect effect was evaluated using 95% bootstrapped confidence intervals.

## Data Availability

The raw data supporting the conclusions of this article will be made available by the authors upon request.

## Abbreviations

The following abbreviations are used in this manuscript:

SMUIS	Social Media Use Integration Scale
SABAS	Smartphone Application-Based Addiction Scale
CESD-10	Center for Epidemiological Studies Depression Scale-10
PCL-5	Posttraumatic Stress Disorder Checklist for DSM-5
BHS-9	Beck Hopelessness Scale-9
STAI-T5	Trait scale of the State-Trait Anxiety Inventory-5
